# Incorporation and solidification mechanism of manganese doped cement clinker

**DOI:** 10.3389/fchem.2023.1165402

**Published:** 2023-04-04

**Authors:** Nan Yang, Aihong Li, Qing Liu, Yanshuai Cui, Zhaojia Wang, Yukun Gao, Jianping Guo

**Affiliations:** ^1^ State Key Laboratory of Solid Waste Reuse for Building Materials, Beijing Building Materials Academy of Science Research, Beijing, China; ^2^ Key Laboratory of Bio-Inspired Smart Interfacial Science and Technology of Ministry of Education, School of Chemistry, Beihang University, Beijing, China

**Keywords:** heavy metals, Raman, XPS, cement kiln co-processing, immobilization

## Abstract

Using municipal and industrial solid waste as a substitute raw material and fuel in cement rotary kiln co-processing is considered an economic and environmentally friendly alternative to the use of traditional fuels. However, the presence of heavy metals in solid waste is a growing concern in the cement rotary kiln co-processing technique. The solidification mechanism of heavy metals in cement clinker is directly related to their stabilization. Cement clinkers doped with manganese oxide (MnO_2_: 0.0%–5.0% wt%) were prepared in a laboratory to investigate the impacts of extrinsic Mn on cement clinker calcination. The insignificant changes in X-ray diffractometer patterns indicated that the fixed Mn had little influence on the mineral lattice structure. Raman spectra and X-ray photoelectron spectroscopy revealed the transformation of the silicate phase when the Mn dose was increased. Moreover, the satisfactory solidification ratio confirmed the incorporation of Mn in the cement clinker. These results provided evidence of the influence rule of Mn in the cement clinker calcination process. Furthermore, Raman spectroscopy showed great potential for the qualitative and semi-quantitative analysis of the cementitious materials derived from cement rotary kiln co-processing. These results will be important for the further development of green cement manufacturing technology.

## Introduction

With the rapid urbanization and infrastructure development in China, the safe disposal of the increasing volumes of municipal and industrial solid waste (SW) is becoming a key environmental issue (Wang et al., 2016; Gong et al., 2022). The heavy metals contained in SW may cause pollution of soil, surface water, and groundwater (Yan et al., 2018; Wang et al., 2022). For example, the accumulation of manganese (Mn) tailings and electrolytic Mn slag have generated environmental and human health problems, even resulting in central nervous system disease unless obtained appropriate treatment after exposure (Wang et al., 2018; Zhang et al., 2021). Cement rotary kiln co-processing of SW is a widely accepted sustainable and economic technique used to dispose of the Mn contained in SW (He et al., 2021; Gong et al., 2022; He et al., 2022; Vaiciene and Simanavicius, 2022). Due to its high calcination temperature and long residence time, the technique is commonly used to fix trace non-volatile harmful ingredients (Zhao et al., 2020). Additionally, the relatively stable alkaline environment in cement kilns can be adapted to manage the complex constituents of SW (Wang et al., 2014; Xu et al., 2019).

Taking advantage of the Mn contained SW as a substitute raw material is considered to be a well-established strategy for the harmless disposal of waste materials and resource utilization (Ukic et al., 2013). Nath and Kumar’s group (Nath and Kumar, 2016) used silico-Mn slag as a component in blended cement, and investigated whether incorporating Mn would lower the reactivity and strength of the cement at the early stage of production. Manganese slag can also be used as a binder and aggregate in Portland cement and concrete, thus improving the mechanical strength and enhancing the properties of the concrete (Nath et al., 2022). In addition to their mechanical influences, the impacts of extrinsic heavy metals on the cement clinker calcination process have attracted much attention recently. It has been reported that a high Mn content promotes the formation of belite that can be incorporated into the mineral phase (Bhatty, 1995). However, the incorporation and lattice substitution mechanism of Mn in clinker remains poorly understood.

There are many analytical instruments available that can characterize the properties of heavy metals in solidified cement materials. The routinely used measurement techniques (e.g., X-ray fluorescence (XRF), X-ray diffraction (XRD), scanning electron microscopy (SEM)) can provide chemical and physical information, such as the chemical composition, morphology, and particle size distribution (Contessi et al., 2021; Polavaram and Garg, 2021). Additionally, X-ray photoelectron spectroscopy (XPS), can be used for the surface characterization of the clinker composition (Kalina et al., 2014; Singh et al., 2020). The binding energy corresponding to specific elements will shift as the chemical bonding environment changes, which provides useful information for elemental analysis (Black et al., 2003). The use of Raman spectroscopy, another powerful analytical technique with non-destructive and ultrasensitive properties, was first demonstrated in cementitious materials/cement chemistry by Bensted (Bensted, 1977). It has since been widely applied in this research area, including monitoring the evolution of hydration products, the Raman imaging of the mineral phase distribution, and *in situ* Raman analysis of cement paste (Black et al., 2006; Garg et al., 2013; Hasanzadeh et al., 2016; Masmoudi et al., 2017; Polavaram and Garg, 2021; Krol et al., 2022). In a recent study, Raman spectroscopy was also used to explore the heavy metals stabilization mechanisms in a cement-stabilized contaminated soil system (Contessi et al., 2021). Raman spectroscopy therefore has an important role in the study of cement clinker phases.

In this study, ordinary clinker doped with manganese dioxide (MnO_2_) was sintered in a laboratory furnace, and the physical chemical properties of clinker doped with 0.0–5.0 wt% MnO_2_ were evaluated. The Raman spectrum was obtained to analyze the mineral phase changes introduced by the addition of Mn. Additionally, the XPS spectrum was used to investigate the chemical environment of Mn, and further explore the heavy metal immobilization mechanism of cementitious materials. In this study, the results provided scientific evidence for the application of Raman spectroscopy in the cement rotary kiln co-processing technique, which will promote the development of green cement manufacturing technology.

## Experimental

### Reagents

Manganese dioxide [MnO_2_, analytical reagent (AR)] and hydrofluoric acid [HF, guaranteed reagent (GR)] were purchased from Aladdin (Shanghai, China). Nitric acid (HNO_3_, GR) and hydrochloric acid (HCl, GR) were purchased from Beijing chemical works (Beijing, China).

### Materials

The cementitious materials in the test consisted of fluorite, sandstone, gypsum, bauxite, and limestone, which were provided by BBMG Liushui Environmental Protection Technology Co., Ltd. cement plant (Beijing, China).

### Characterization

The chemical characterization was determined by XRF (Axios Max, PANalytical, Holland). The mineralogical phases were identified by XRD (Ultima IV, Rigaku, Japan) using 2θ angle range of 5.0°–80° with a scan rate of 0.2 s per step and a step size of 0.02. The CuKα radiation (=1.5418 Å) was generated at 40 kV and 40 mA. The heavy metals were measured by Inductively Coupled Plasma-Mass Spectrometry (ICP-MS, NexION 300X, Perkin Elmer, United States).

Raman spectra were recorded with a JY HR800 Raman spectrometer (HORIBA, Japan), equipped with a ×50 objective lens (numerical aperture (NA) = 0.5) and a He−Ne laser with 514 and 633 nm wavelength. The laser power values measured in the experiments were obtained from a power meter (0.75 mW at the samples with a spot area of approximately 1.4 μm^2^). The Raman band of a silicon wafer at 520.8 cm^−1^ was used to calibrate the spectrometer. The data integration time was 5 s for two accumulations.

The XPS analysis was carried out using an AXIS Supra X-ray photoelectron spectrometer with a monochromatic Al Kα (hν = 1486.7 eV) X-ray source operating at 225 W. The XPS spectra were measured under a 2 × 10^−8^ Pa vacuum, and the high resolution spectra were measured with a step size of 0.1 eV and pass energy of 20 eV. A standard Shirley background was used for all analyses. The binding energies were calibrated using the adventitious carbon peak at 284.8 eV.

### Preparation of the Mn doped clinker

Manganese ions were introduced into the raw materials (RMs) in the form of MnO_2_ at doses of 0.0, 0.5, 1.0, 2.0, 3.0, 4.0, and 5.0 wt%, which were referred to as CM-Blank, CM-0.5%, CM-1.0%, CM-2.0%, CM-3.0%, CM-4.0%, and CM-5.0%, respectively. The composition of the cement designed for the study is presented in Table 1. All materials were ground in a vibrating ball mill for 10 min and then the powder was pressed into cylindrical blocks (diameter = 7 mm, height = 5 mm) at 100 kN to ensure a more uniform clinkering process. The cylindrical blocks were placed in a corundum crucible and heated up to 1450°C at a rate of 15°C/min, sintering for 30 min. The resulting clinkers were cooled rapidly outside the furnace and ground as homogenized clinker material (CM) powder for further analysis.

**TABLE 1:** The composition of the cement raw materials.

	Limestone (%)	Sandstone (%)	Gypsum (%)	Fluorite (%)	Bauxite (%)	Mn (%)
CM-Blank	65.5	5.0	12.0	1.5	16.0	-
CM-0.5%	65.2		11.5			0.5
CM-1%	64.9		11.0			1
CM-2%	63.8		10.0			2
CM-3%	62.8		11.5			3
CM-4%	61.7		11.0			4
CM-5%	60.6		10.0			5

## Results and discussion

### Characterization of clinker

The chemical composition of the CM is shown in Table 2. The major constituents were oxides of calcium (Ca), aluminum (Al), and silicon (Si), The total wt% of CaO + Al_2_O_3_ + SiO_2_ were 84.50%, 82.87%, 81.70%, 80.39%, 78.42%, 78.01%, and 75.88% in the blank CM sample and 0.5–5% Mn doped clinker samples, respectively.

**TABLE 2:** Composition of the Mn doped clinker material samples.

Compound	Mass fraction (%)						
	CM-blank	CM-0.5%	CM-1%	CM-2%	CM-3%	CM-4%	CM-5%
CaO	54.19	53.25	51.63	50.22	49.81	48.87	47.61
Al_2_O_3_	18.06	17.53	17.47	17.78	16.80	16.99	16.57
SiO_2_	12.25	12.10	12.60	12.38	11.80	12.16	11.70
SO_3_	10.76	11.40	12.09	11.94	12.07	11.18	11.35
MnO_2_	0.00	0.98	1.90	3.40	5.10	6.79	8.50
MgO	2.24	2.29	1.82	1.86	1.99	1.65	1.80
Fe_2_O_3_	0.85	0.93	0.96	0.86	0.83	0.87	0.99
TiO_2_	0.80	0.80	0.84	0.79	0.73	0.84	0.70
K_2_O	0.29	0.29	0.24	0.28	0.26	0.24	0.26
SrO	0.11	0.11	0.13	0.11	0.12	0.11	0.11
Cl	0.10	0.10	0.09	0.09	0.07	0.10	0.08
L.O.I. of RM	30.20%	29.82%	29.07%	28.22%	27.87%	27.58%	27.19%

**FIGURE 1:**
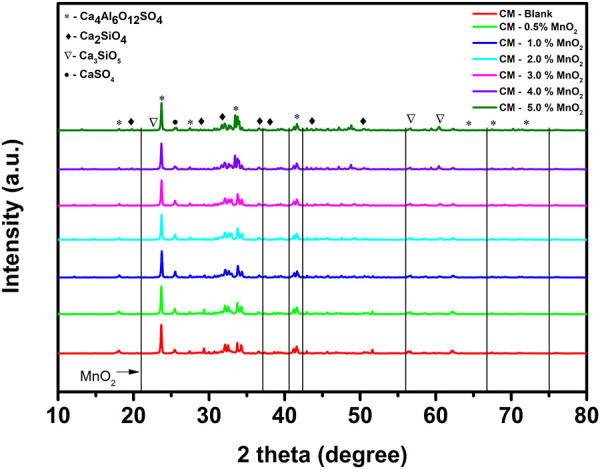
The XRD patterns of Mn doped clinker samples (red: CM-blank, green: CM-0.5% MnO_2_, blue: CM-1% MnO_2_, cyan: CM-2% MnO_2_, magenta: CM-3% MnO_2_, violet: CM-4% MnO_2_, olive: CM-5% MnO_2_, black: reference diffraction pattern of MnO_2_).

The XRD patterns of Mn doped clinker samples are shown in Figure 1. The major component was Ca_4_Al_6_O_12_SO_4_. The characteristic peaks of Ca_2_SiO_4_ and Ca_3_SiO_5_ indicated the formation of a clinker mineral phase after the high temperature clinker calcination process. The XRD results were consistent with the data in Table 2, i.e., the dominant elements were Ca, Al, and Si. When the XRD results of the blank clinker sample were compared with those for Mn doped clinker samples, no new phases or obvious changes of crystal structure were apparent. Moreover, the referenced diffraction pattern of MnO_2_ also did not exist in the clinker, even when the MnO_2_ dose was 5.0%. It was therefore speculated that the Mn ion might be incorporated into the crystal lattice, and was unable to change the clinker crystal structure.

### Leaching test and solidification ratio analysis

The leaching content of Mn in CM was determined according to Chinese Standard GB/T 30810-2014 (Test methods for leachable ions of HMs in cement mortar) and the results are shown in Table 3 (General Administration of Quality Supervision I a Q, P.R. China, 2014). The solidification ratio was calculacated to evaluate the retention of heavy metals in the clinker samples after cement clinker calcination process. High solidification ratio indicates high content of heavy metals solidified in clinker and has low risk to environment pollution (Gao et al., 2017). The solidification ratios of 0.5%–5% Mn doped clinker samples was calculated from the ICP-MS results and were 83.84%, 87.43%, 84.29%, 82.04%, 83.97%, and 82.62%, respectively (Miao, 2018). The solidification ratios calculated according to the XRF results were 86.89%, 85.19%, 77.17%, 77.52%, 77.69%, and 78.21%, respectively. The solidification ratio of Mn in cement was high when the Mn content was 0.5% and 1%, which indicated the effective fixation in cement clinker. The solidification ratio was lower when the Mn dose exceeded 1%. In general, the satisfactory solidification ratio provided evidence of the immobilization of Mn in the formation of cementitious materials.

**TABLE 3:** Leaching test and solidification ratio of the Mn doped clinker material samples.

Sample	L.O.I. of RM %	Content (g/Kg)	Solidification ratio (ICP) %	Content %	Solidification ratio (XRF) %
CM-BLANK	30.20	0.08	-	0.00	-
CM-0.5%	29.82	6.05	83.84	0.62	86.89
CM-1%	29.07	12.41	87.43	1.20	85.19
CM-2%	28.22	23.57	84.29	2.15	77.17
CM-3%	27.87	34.20	82.04	3.22	77.52
CM-4%	27.58	46.46	83.97	4.29	77.69
CM-5%	27.19	56.82	82.62	5.37	78.21

### Raman analysis of Mn doped clinker samples

**FIGURE 2:**
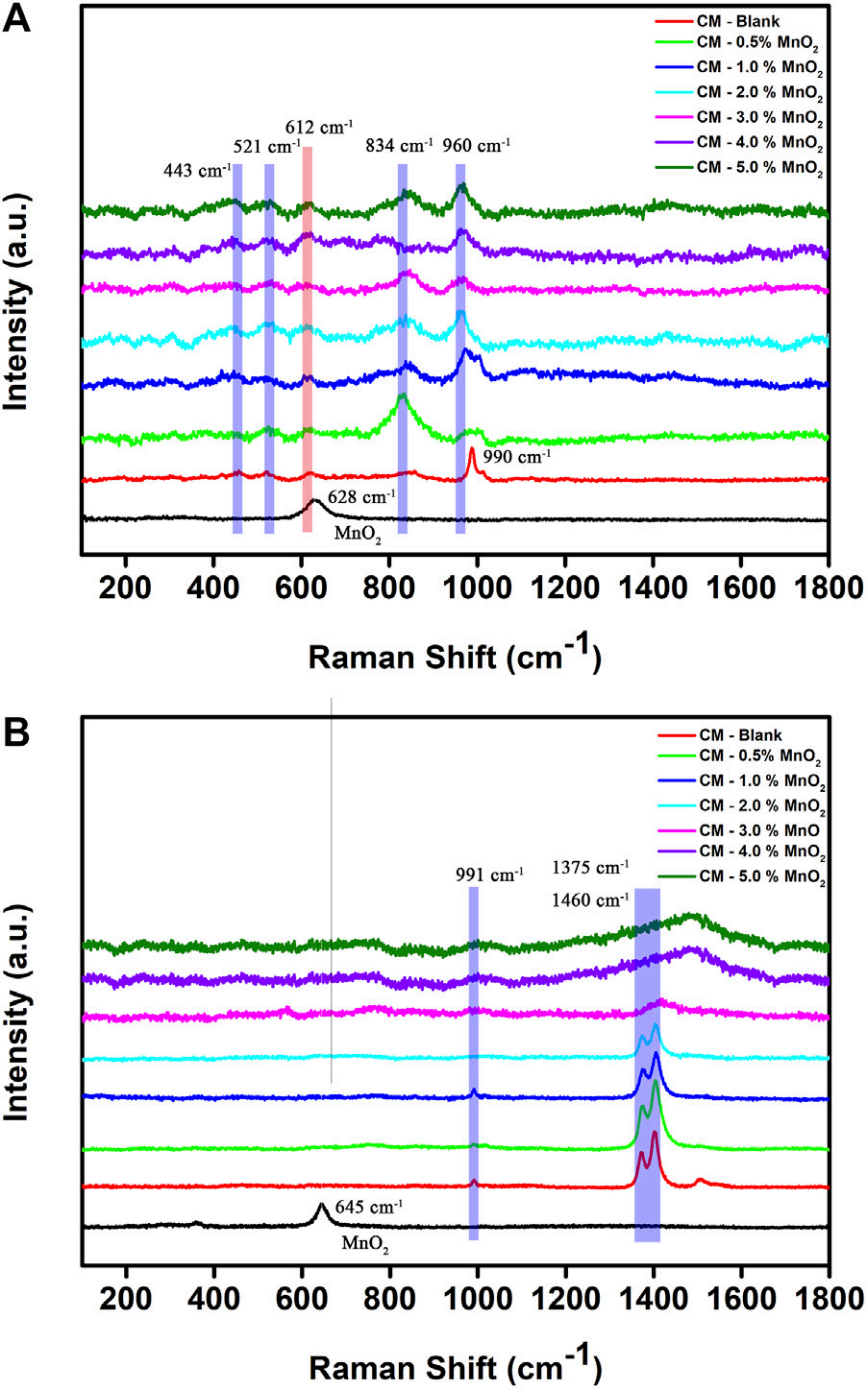
Raman spectra of Mn doped clinker samples recorded with **(A)** 514 nm and **(B)** 633 nm laser excitation (red: CM-blank, green: CM-0.5% MnO_2_, blue: CM-1% MnO_2_, cyan: CM-2% MnO_2_, magenta: CM-3% MnO_2_, violet: CM-4% MnO_2_, olive: CM-5% MnO_2_, black: MnO_2_).

Figure 2A shows the Raman spectra of Mn doped clinker samples recorded with 514 nm laser excitation. The signals at 443 and 612 cm^−1^ were due to the υ2 and υ4 bending from [SO_4_]^2−^ groups, the peak at 521 cm^−1^ was due to the υ1 [AlO_4_]^5−^ tetrahedra in belite, and the 834 cm^−1^ band was assigned to the υ1 [SiO_4_]^4−^ symmetric stretching in alite (Kloprogge et al., 2002; Potgieter-Vermaak et al., 2006; Black and Brooker, 2007; Black, 2009). The signals below 300 cm^−1^ were assigned to Ca-O bonds (Torrens-Martin et al., 2013). A sharp band at 990 cm^−1^ was assigned to the υ1 [SO_4_] ^2−^ vibrations in the blank CM sample. However, the band weakened and disappeared when the Mn dose was 2%, and there was a band formed at 960 cm^−1^ as the Mn content increased. This phenomenon may be due to the mineral phase transformation from yeelimite to belite, which was caused by the addition of Mn. Moreover, the peak at 628 cm^−1^ that was assigned to MnO_2_ was not found in clinker samples, proving the solidification effect of Mn after the high temperature calcination in the mineral formation process.

For 633 nm laser excitation (Figure 2B), the band at 991 cm^–1^, which corresponded to yeelimite, disappeared in the 2% Mn doped clinker sample. Two major characteristic peaks could be observed at 1375 cm^–1^ and 1406 cm^–1^ when the dosage of Mn was below 3%, which could be assigned to vibrations of silicate materials (Hasanzadeh et al., 2016). Those two peaks became broader and red-shifted as the Mn content increased, which indicated a poor crystalline phase of the CM. The impurities in clinker mineral lattice and disordered silicates, aluminosilicates minerals may result in fluorescence emissions and background which became a big challenge to obtaining true Raman bands (Dyer et al., 1993; Bonen et al., 1994; Newman et al., 2005). The foreign Mn ions present in the clinker samples at a content exceeding 3% may broaden the spectra and cause a mineral phase disorder of the cementitious materials. Although the Mn content exceeded 3%, the characteristic MnO_2_ peak was still not found in CM samples.

Overall, the increased addition of Mn influenced the crystallinity of the clinker; however, the Mn ion could be effectively immobilized and fixed in cementitious materials according to the leaching solidification ratio, XRD, and Raman spectroscopy results.

### The XPS analysis of Mn doped clinker samples

**FIGURE 3:**
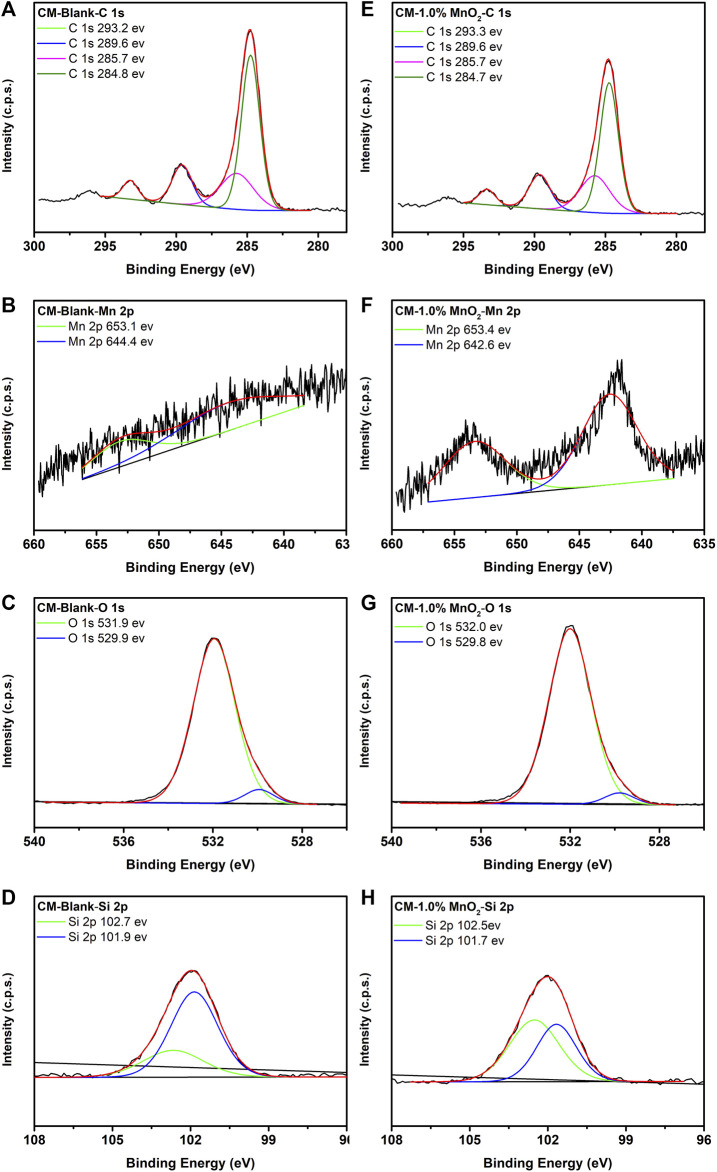
The XPS spectra of C 1s, Mn 2p, O 1s, and Si 2p for blank **(A−D)** and 1% Mn doped CM samples **(E−H)**.

The chemical state and constituent composition of clinker samples were evaluated by XPS. The XPS narrow spectra of C 1s, Mn 2p, O 1s, and Si 2p for blank and 1% Mn doped CM samples are shown in Figure 3, and the spectra of the other Mn doped CM samples are provided in Supplementary Figure S1. The C 1s peaks identified at 284.8, 285.7, and 289.6 eV were the binding energies of contamination adventitious carbon, which accounted for the C-C, C-O-C, and O-C=O components, respectively (Kalina et al., 2014; Singh et al., 2020). The bonding energy situated at 293.2 eV accounted for C-F, which may form due to the presence of fluorite in the RMs. There were two peaks identified at 642.6 and 653.4 eV in Mn 2p, which corresponded to Mn-O in MnO_2_. In the O 1s spectrum, the 529.8 eV peak energy was attributed to Ca-O and Mn-O, and the 532.0 eV peak energy was attributed to Si-O (Wang et al., 2016). The typical peak at 102.7–102.5 eV was assigned to SiO_2_, and the peak at 101.9–101.7 eV was assigned to Si in belite (Estokova et al., 2018; Singh et al., 2020). The other O 1s spectrum for Mn doped CM samples is provided in Supplementary Figure S2.

**FIGURE 4:**
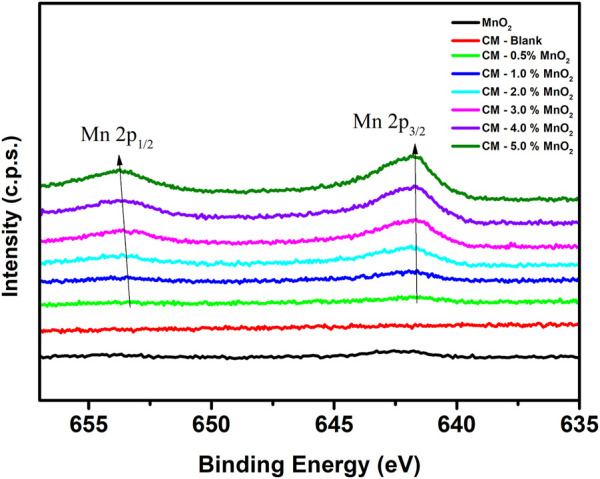
The stacked XPS spectra of Mn 2p for MnO_2_ and Mn doped clinker samples (red: CM-blank, green: CM-0.5% MnO_2_, blue: CM-1% MnO_2_, cyan: CM-2% MnO_2_, magenta: CM-3% MnO_2_, violet: CM-4% MnO_2_, olive: CM-5% MnO_2_, black: MnO_2_).

Figure 4 shows the stacked Mn 2p spectra of MnO_2_ and Mn doped clinker samples. For the Mn doped CM samples, it was found that the Mn 2p peak shifted to a higher bonding energy as the Mn dose increased. The changes of the chemical bonding environments can result in shifts in photoelectron energy, thus the drift of the Mn 2p peak was accordingly speculated as the changes in bonding structure (Black et al., 2003; Estokova et al., 2018). The Mn 2p peak shifted to a higher bonding energy indicating the progressive disordering of the silicate structure which may cause by the incorporation effect of silicate and/or aluminosilicate towards Mn (Rheinheimer and Casanova, 2014).

Furthermore, Supplementary Figure S3 shows the transformation of the Si 2p spectra. Compared with a blank sample, the peak proportion of 102.7 eV increased but was lower than that of 101.8 eV in the 0.5% Mn doped CM sample. As the Mn content increased, the peak proportions of the two peaks were the same as in the 1% Mn doped CM sample, but the peak proportion at 102.7 eV was higher than at 101.8 eV when the Mn content was 2%. This trend stabilized in the 3%–5% Mn doped CM samples. This phenomenon of the two peaks reflected the transformation of the SiO_2_ and belite phases, which could explain the optimal solidification ratio of the 1% Mn doped CM sample shown in Table 3.

This change in the Si 2p spectra was hypothesized to be caused by the differences in the chemical environment of Mn doped CM samples, which was caused by the solidification of Mn in clinker (Nath et al., 2022). The addition of Mn influenced the formation of the silicate phase and the increasing Mn content generated an increase in the SiO_2_ content and reduction in the belite phase, which may cause a disordered crystal structure. However, the XRD pattern and the solidification ratio were still satisfactory when the Mn content was 5%.

## Conclusion

In this study, CMs doped with 0.0%–5.0% Mn were sintered in a laboratory. The physical and chemical properties of samples were evaluated by XRF, XRD, Raman spectroscopy, and XPS. The changes in the XRD patterns were insignificant due to the homologous lattice structure, which confirmed the solidification of Mn in the clinker. The Raman spectrum revealed the transformation of the belite phase with the addition of Mn. Moreover, the variation of the XPS spectrum of Mn 2p and Si 2p indicated a change in the chemical environment in CM samples as the Mn dose increased. Overall, although the addition of Mn can influence the silicate crystalline structure, a satisfactory solidification ratio was achieved following the incorporation of 0.5%–5.0% Mn in cementitious materials. In summary, the results confirmed the usefulness of Raman spectroscopy as a qualitative and semi-quantitative analysis method in the field of cementitious materials, which will promote the development of green cement manufacturing technology.

## Data Availability

The original contributions presented in the study are included in the article/Supplementary Material, further inquiries can be directed to the corresponding authors.

## References

[B1] Bensted J. (1977). Raman spectral studies of carbonation phenomena. Cem. Concr. Res. 7 (2), 161–164. 10.1016/0008-8846(77)90026-6

[B2] Bhatty J. (1995). Role of minor elements in cement manufacture and use. United States: Portland Cement Association.

[B3] Black L. Breen C. Yarwood J. Phipps J. Maitland G. (2006). *In situ* Raman analysis of hydrating C(3)A and C(4)AF pastes in presence and absence of sulphate. Adv. Appl. Ceram. 105 (4), 209–216. 10.1179/174367606x120179

[B4] Black L. Brooker A. (2007). SEM-SCA: Combined SEM - Raman spectrometer for analysis of OPC clinker. Adv. Appl. Ceram. 106 (6), 327–334. 10.1179/174367607x228052

[B5] Black L. Garbev K. Stemmermann P. Hallam K. R. Allen G. C. (2003). Characterisation of crystalline C-S-H phases by X-ray photoelectron spectroscopy. Cem. Concr. Res. 33 (6), 899–911. 10.1016/s0008-8846(02)01089-x

[B6] Black L. (2009). Spectroscopic properties of inorganic and organometallic compounds: Volume 40. London: The Royal Society of Chemistry, 72–127.

[B7] Bonen D. Johnson T. J. Sarkar S. L. (1994). Characterization of principal clinker minerals by FT-Raman microspectroscopy. Cem. Concr. Res. 24 (5), 959–965. 10.1016/0008-8846(94)90016-7

[B8] Contessi S. Dalconi M. C. Pollastri S. Calgaro L. Meneghini C. Ferrari G. (2021). Cement-stabilized contaminated soil: Understanding Pb retention with XANES and Raman spectroscopy. Sci. Total Environ. 752, 141826. 10.1016/j.scitotenv.2020.141826 32889270

[B9] Dyer C. D. Hendra P. J. Forsling W. (1993). The Raman spectroscopy of cement minerals under 1064 nm excitation. Spectrochim. Acta Part A Mol. Spectrosc. 49 (5), 715–722. 10.1016/0584-8539(93)80094-q

[B10] Estokova A. Palascakova L. Kanuchova M. (2018). Study on Cr (VI) leaching from cement and cement composites. IJERPH 15 (4), 824. 10.3390/ijerph15040824 29690550 PMC5923866

[B11] Gao J. Ni W. Yu M. Xu C. Gao H. (2017). Study on calcination cement clinker using a lead-zinc tailings as raw. Mater. Metal. mine (3), 192–196.

[B12] Garg N. Wang K. J. Martin S. W. (2013). A Raman spectroscopic study of the evolution of sulfates and hydroxides in cement-fly ash pastes. Cem. Concr. Res. 53, 91–103. 10.1016/j.cemconres.2013.06.009

[B13] General Administration of Quality Supervision I a Q, P.R. China (2014). Test methods for leachable ions of heavy metals in cement mortar.

[B14] Gong J. Yu L. L. Li Z. P. Shi X. M. (2022). Mechanical activation improves reactivity and reduces leaching of municipal solid waste incineration MSWI bottom ash in cement hydration system. J. Clean. Prod. 363, 132533. 10.1016/j.jclepro.2022.132533

[B15] Hasanzadeh B. Liu F. J. Sun Z. H. (2016). Monitoring hydration of UHPC and conventional paste by quantitative analysis on Raman patterns. Constr. Build. Mater. 114, 208–214. 10.1016/j.conbuildmat.2016.03.178

[B16] He S. C. Jiang D. Y. Hong M. H. Liu Z. H. (2021). Hazard-free treatment and resource utilisation of electrolytic manganese residue: A review. J. Clean. Prod. 306, 127224. 10.1016/j.jclepro.2021.127224

[B17] He W. L. Li R. Zhang Y. Nie D. P. (2022). Synergistic use of electrolytic manganese residue and barium slag to prepare belite-sulphoaluminate cement study. Constr. Build. Mater. 326, 126672. 10.1016/j.conbuildmat.2022.126672

[B18] Kalina L. Masilko J. Koplik J. Soukal F. (2014). XPS characterization of polymer-monocalcium aluminate interface. Cem. Concr. Res. 66, 110–114. 10.1016/j.cemconres.2014.07.021

[B19] Kloprogge J. T. Schuiling R. D. Ding Z. Hickey L. Wharton D. Frost R. L. (2002). Vibrational spectroscopic study of syngenite formed during the treatment of liquid manure with sulphuric acid. Vib. Spectrosc. 28 (2), 209–221. 10.1016/s0924-2031(01)00139-4

[B20] Krol M. Kolezynski A. Florek P. Jele P. Kozie D. Mozgawa W. (2022). Full spectroscopic characterization of clinker minerals (anhydrous cement). J. Mol. Struct. 1255, 132454. 10.1016/j.molstruc.2022.132454

[B21] Masmoudi R. Kupwade-Patil K. Bumajdad A. Buyukozturk O. (2017). *In situ* Raman studies on cement paste prepared with natural pozzolanic volcanic ash and Ordinary Portland Cement. Constr. Build. Mater. 148, 444–454. 10.1016/j.conbuildmat.2017.05.016

[B22] Miao X. (2018). The study of the effect of cement kiln co-disposal technology on curing heavy metals. Zhong Guo Shui Ni (7), 84–86.

[B23] Nath S. K. Kumar S. (2016). Evaluation of the suitability of ground granulated silico-manganese slag in Portland slag cement. Constr. Build. Mater. 125, 127–134. 10.1016/j.conbuildmat.2016.08.025

[B24] Nath S. K. Randhawa N. S. Kumar S. (2022). A review on characteristics of silico-manganese slag and its utilization into construction materials. Resour. Conserv. Recycl. 176, 105946. 10.1016/j.resconrec.2021.105946

[B25] Newman S. P. Clifford S. J. Coveney P. V. Gupta V. Blanchard J. D. Serafin F. (2005). Anomalous fluorescence in near-infrared Raman spectroscopy of cementitious materials. Cem. Concr. Res. 35 (8), 1620–1628. 10.1016/j.cemconres.2004.10.001

[B26] Polavaram K. C. Garg N. (2021). Enabling phase quantification of anhydrous cements via Raman imaging. Cem. Concr. Res. 150, 106592. 10.1016/j.cemconres.2021.106592

[B27] Potgieter-Vermaak S. S. Potgieter J. H. Van Grieken R. (2006). The application of Raman spectrometry to investigate and characterize cement, Part I: A review. Cem. Concr. Res. 36 (4), 656–662. 10.1016/j.cemconres.2005.09.008

[B28] Rheinheimer V. Casanova I. (2014). An X-ray photoelectron spectroscopy study of the hydration of C2S thin films. Cem. Concr. Res. 60, 83–90. 10.1016/j.cemconres.2014.03.005

[B29] Singh S. Dalbehera M. M. Rawat A. Sharma P. (2020). Carbonation study and determination of cement to sand ratio in hardened cement mortar by X-ray photoelectron spectroscopy. Surf. Interface Anal. 52 (10), 603–610. 10.1002/sia.6797

[B30] Torrens-Martin D. Fernandez-Carrasco L. Martinez-Ramirez S. (2013). Hydration of calcium aluminates and calcium sulfoaluminate studied by Raman spectroscopy. Cem. Concr. Res. 47, 43–50. 10.1016/j.cemconres.2013.01.015

[B31] Ukic S. Dimic P. Siljeg M. Bosnjak M. U. Sipusic J. Bolanca T. (2013). Manganese waste mud immobilization in cement natural zeolite lime blend: Process optimization using artificial neural networks and multi-criteria functions. Materwiss. Werksttech. 44 (4), 273–281. 10.1002/mawe.201300050

[B32] Vaiciene M. Simanavicius E. (2022). The effect of municipal solid waste incineration ash on the properties and durability of cement concrete. Materials 15 (13), 4486. 10.3390/ma15134486 35806610 PMC9267427

[B33] Wang F. Z. Shang D. C. Wang M. G. Hu S. G. Li Y. Q. (2016). Incorporation and substitution mechanism of cadmium in cement clinker. J. Clean. Prod. 112, 2292–2299. 10.1016/j.jclepro.2015.09.127

[B34] Wang H. Q. Zhang S. G. Wu B. N. (2018). Experimental study on selection of early-strength agent for low-strength cementitious materials prepared with manganese tailings. Environ. Earth Sci. 77 (6), 231. 10.1007/s12665-018-7415-5

[B35] Wang L. Huang X. Y. Li X. T. Bi X. T. Yan D. H. Hu W. Z. (2022). Simulation of heavy metals behaviour during Co-processing of fly ash from municipal solid waste incineration with cement raw meal in a rotary kiln. J. Waste Manag. 144, 246–254. 10.1016/j.wasman.2022.03.031 35413523

[B36] Wang X. Liu C. Yan B. Zhen X. Zhang J. Wang H. (2014). Statue and application of abroad and home co-processing of municipal solid waste by cement kiln. Gui Suan Yan Tong Bao 33 (8), 1989–1995.

[B37] Xu J. C. Ping L. Cao H. H. Liu W. Gu Y. H. Lin X. H. (2019). Application status of co-processing municipal sewage sludge in cement kilns in China. Sustainability 11 (12), 3315. 10.3390/su11123315

[B38] Yan D. Peng Z. Yu L. Sun Y. Yong R. Helge Karstensen K. (2018). Characterization of heavy metals and PCDD/Fs from water-washing pretreatment and a cement kiln co-processing municipal solid waste incinerator fly ash. J. Waste Manag. 76, 106–116. 10.1016/j.wasman.2018.03.006 29573924

[B39] Zhang S. H. Li X. D. Zhang W. Fan F. Wang Y. Z. (2021). Study on the leaching toxicity and performance of manganese slag-based cementitious materials. Mater. Res. Express 8 (12), 125308. 10.1088/2053-1591/ac4329

[B40] Zhao X. Rui W. Xu S. Zhang G. Ji L. (2020). Research progress on solidification of heavy metals during co-processing of hazardous waste in cement kilns. Shui Ni Gong Chen (1), 79–82.

