# Supplementary material for: Incorporation and solidification mechanism of manganese doped cement clinker
Source: Front Chem. 2023 Apr 4;11:1165402. doi: 10.3389/fchem.2023.1165402 (PMC10110877; doi:10.3389/fchem.2023.1165402)
Supplement: Supplementary file 1 [file DataSheet1.docx]

Incorporation and solidification mechanism of manganese doped cement clinker

# XPS analysis of Mn doped clinker samples


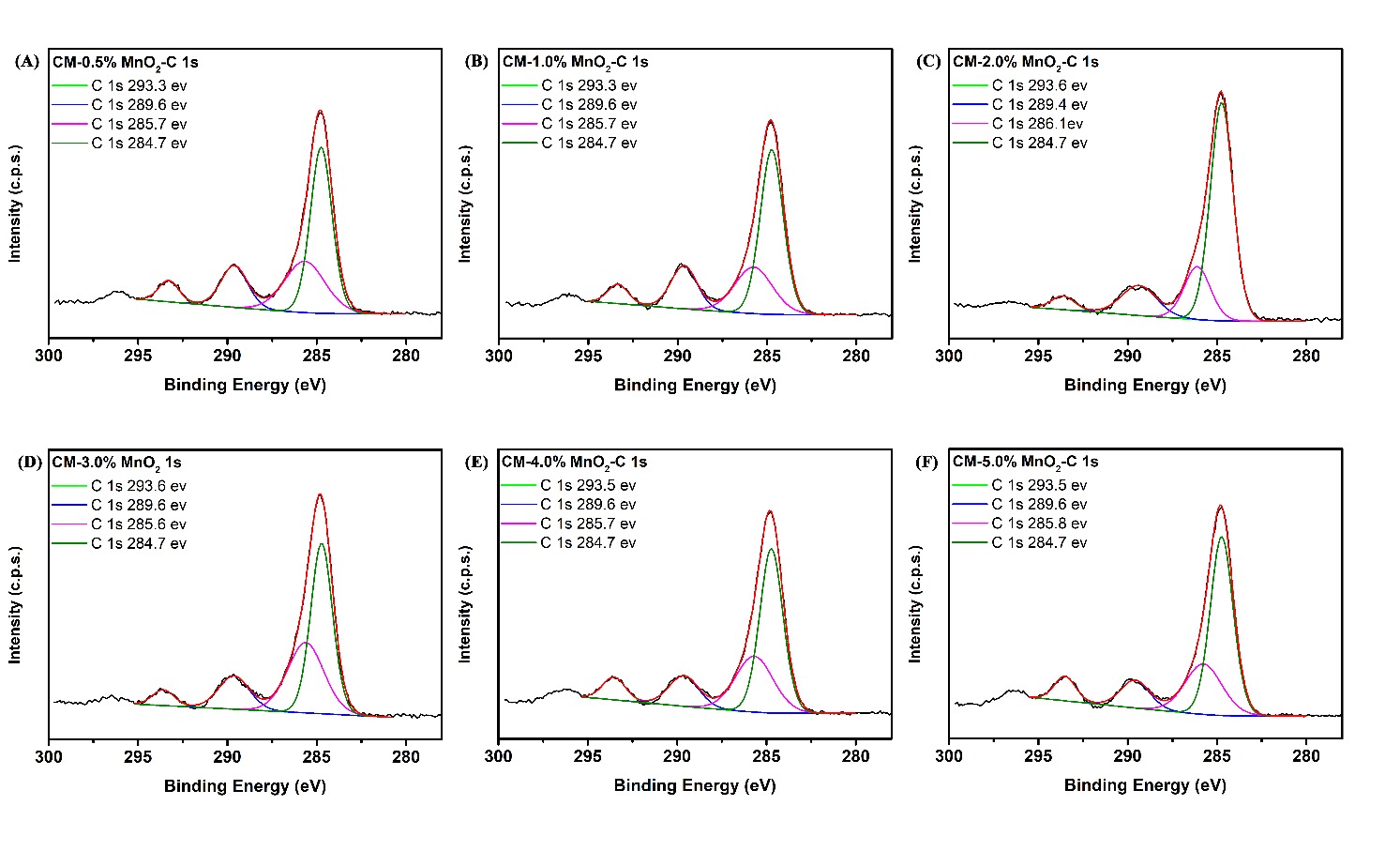


**Supplementary Figure 1.** The XPS spectra of C 1s for Mn doped clinker samples ((A): CM-0.5% MnO_2_, (B): CM-1% MnO_2_, (C): CM-2% MnO_2_, (D): CM-3% MnO_2_, (E): CM-4% MnO_2_, (F): CM-5% MnO_2_).


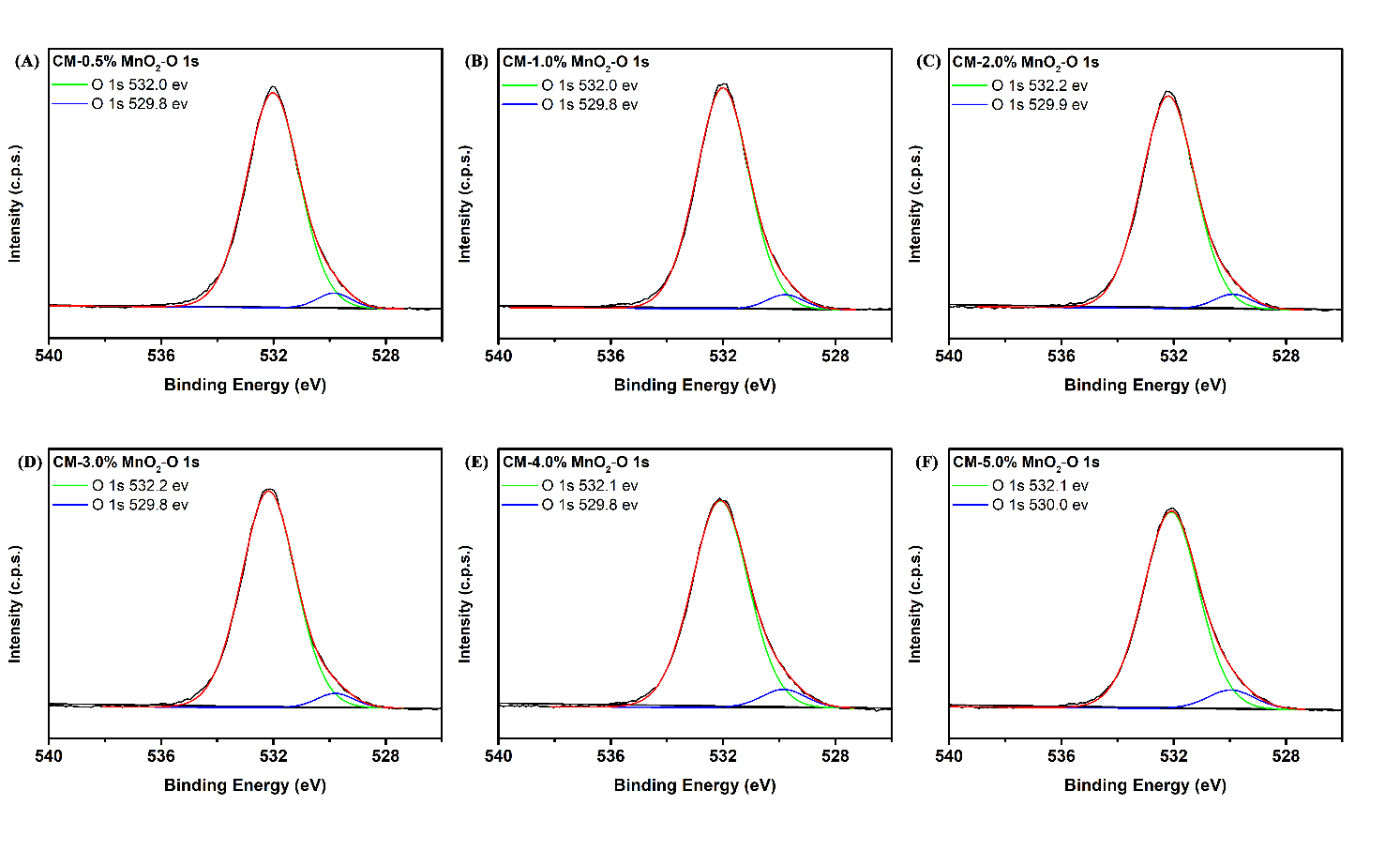


**Supplementary Figure 2.** The XPS spectra of O 1s for Mn doped clinker samples ((A): CM-0.5% MnO_2_, (B): CM-1% MnO_2_, (C): CM-2% MnO_2_, (D): CM-3% MnO_2_, (E): CM-4% MnO_2_, (F): CM-5% MnO_2_).


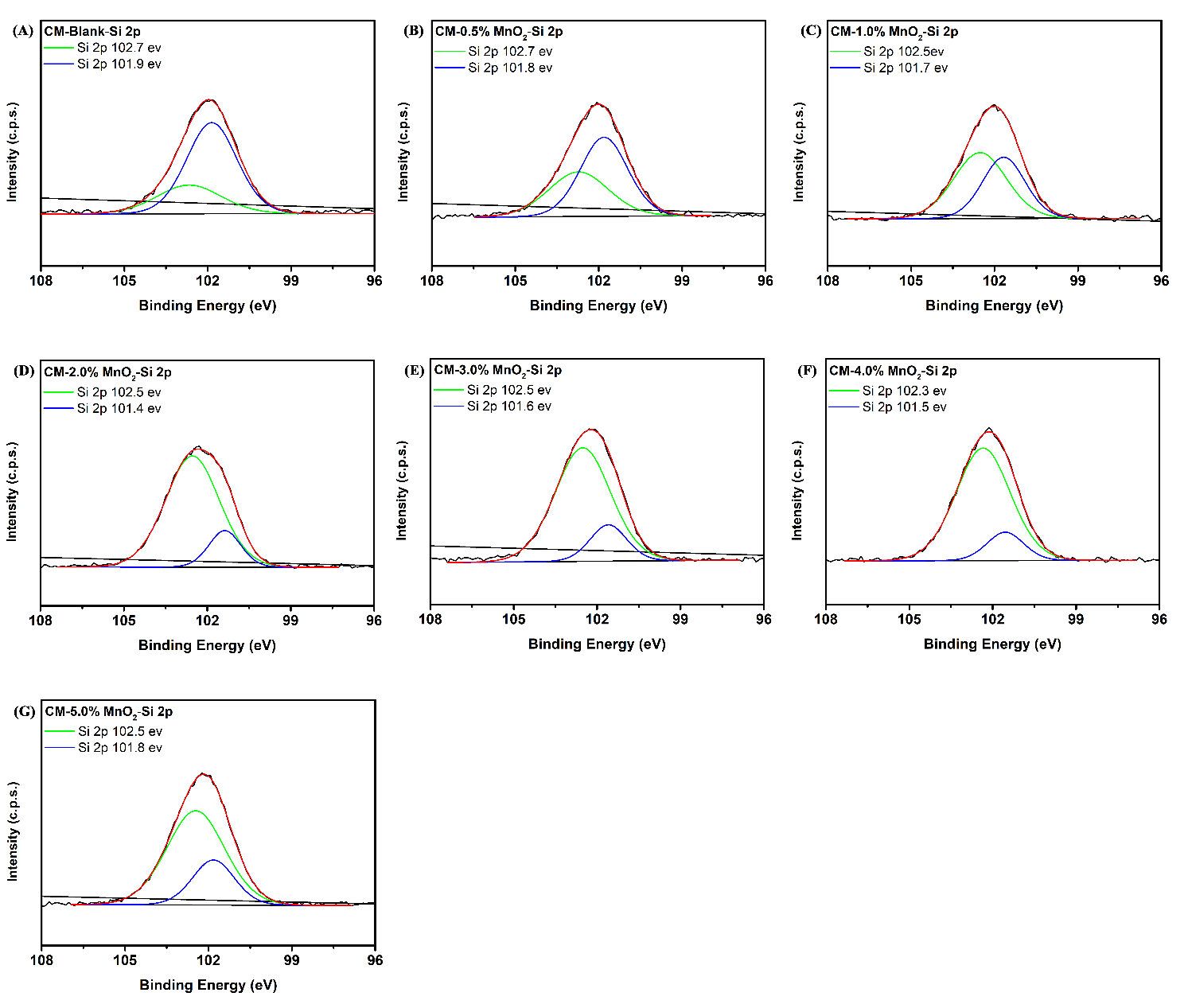


**Supplementary Figure 3.** The XPS spectra of Si 2p for Mn doped clinker samples ((A): CM-blank, (B): CM-0.5% MnO_2_, (C): CM-1% MnO_2_, (D): CM-2% MnO_2_, (E): CM-3% MnO_2_, (F): CM-4% MnO_2_, (G): CM-5% MnO_2_).
